# HMGR overexpression and interference affect the expression of steroidogenic genes and cholesterol content in bovine intramuscular adipocytes

**DOI:** 10.1038/s41598-020-73626-8

**Published:** 2020-10-06

**Authors:** Haichao Lin, Chen Wei, Xianglun Zhang, Wei You, Qing Jin, Xiuwen Tan, Hongbo Zhao, Chen Zhang, Xiaomu Liu, Guifen Liu

**Affiliations:** 1 Institute of Animal Science and Veterinary Medicine, Shandong Academy of Agricultural Sciences, Lichen Region, Jinan, 250100 China; 2 Shandong Key Lab of Animal Disease Control and Breeding, Jinan, 250100 China; 3 Shandong Provincial Testing Center of Beef Cattle Performance, Jinan, 250100 China; 4 College of Life Sciences, Shandong Normal University, Jinan, 250100 China

**Keywords:** Genetics, Molecular biology

## Abstract

Previously, we found that mevalonic acid stimulates 3-hydroxy-3-methyl-glutaryl-coenzyme A reductase (HMGR) expression in bovine intramuscular adipocytes to influence adipocyte differentiation. However, any direct links among HMGR, steroidogenic genes, and cholesterol content remain unclear. RNA-Seq was conducted to determine the differences between the gene expression profiles of bovine adipocytes containing different HMGR expression constructs. In total, 10,234 differentially expressed genes (DEGs) were found. Of these, 35 and 6 DEGs between the control and the overexpression groups were functionally related to lipid and energy metabolism, respectively. In addition, 43 and 8 DEGs between the control and the HMGR inhibition groups were related to lipid and energy metabolism, respectively. Several DEGs related to lipid and energy metabolism were also identified between the HMGR overexpression group and the HMGR interference group, and many DEGs were correlated positively or negatively with the overexpression or inhibition of HMGR. We also found that, following the activation or inhibition of the HMGR gene, AMP-activated protein kinase (AMPK) and sirtuin type 1 (SIRT1) had opposite expression patterns in bovine intramuscular adipocytes. Interestingly, the HMGR gene was downregulated when HMGR was overexpressed, and upregulated when HMGR was inhibited. Our findings establish a theoretical understanding of signaling pathways involved in cholesterol synthesis by elucidating the relationships between key genes.

## Introduction

Intramuscular fat (IMF) is an important factor influencing meat quality in beef cattle^1–4^. The right amount of IMF can enhance meat quality traits such as flavor, juiciness, and tenderness^5,6^. One of the important traits in beef that researchers have focused on are the mechanisms and regulation of fat deposition in fat tissue following increased or decreased fat intake, which might be related to concentrations of circulating cholesterol^7,8^. With increasing concerns regarding the relationship between fat intake and health, consumers have become more conscious of what constitutes a healthy diet, and are increasingly demanding products with reduced cholesterol content^9^. Due to the high cholesterol content and the appealing taste of IMF, beef cattle might be a suitable candidate for cholesterol reduction and dietary improvement.

Three-hydroxy-3-methyl-glutaryl-coenzyme A reductase (HMGR), is a rate-limiting enzyme of cholesterol synthesis. This protein can catalyze the conversion of 3-hydroxy-3-methyl-glutaryl-coenzyme A (HMG-CoA) to mevalonate and is a key factor in the regulation of cholesterol content in *vivo*^10,11^. Sirtuin type 1 (SIRT1), AMP-activated protein kinase (AMPK), and lipoprotein lipase (LPL) play important roles in the regulation of energy metabolism. Previous studies have shown that long-term moderate caloric restriction and resveratrol supplementation can improve lipid disorders and decrease fat accumulation by regulating the SIRT1-autophagy pathway^12^. However, it remains unclear whether there is any direct link among HMGR, steroidogenic genes, and cholesterol content.

Cholesterol content is a complex genetic trait. The genetics underlying the biochemical process and molecular background that determine cholesterol content are not yet fully understood, particularly with regard to local Chinese cattle breeds. Using RNA-Seq analysis to select differently expressed genes (DEGs) correlated with cholesterol levels, the present study aimed to explore the effects of HMGR overexpression and interference on the expression patterns of cholesterol-related genes, as well as on differential gene expression in bovine intramuscular adipose cells.

## Results

### Adipose cell morphology and lipid accumulation

**Figure 1:**
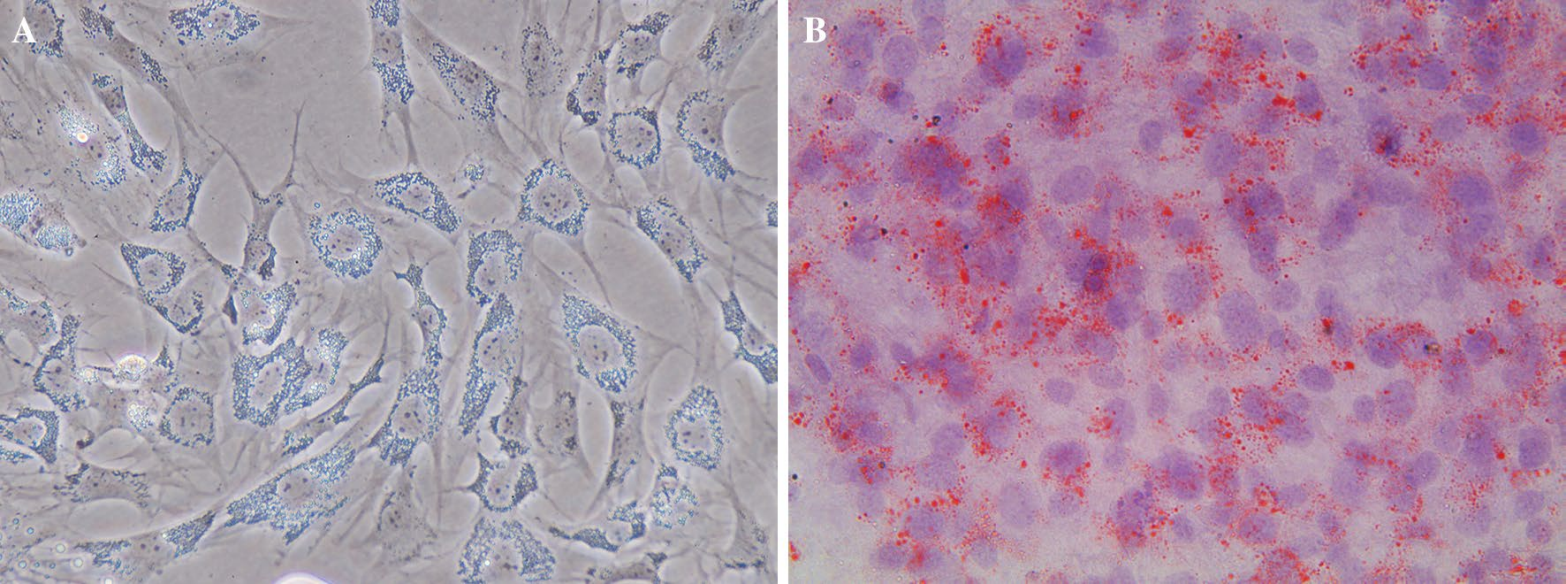
(**A**) The bovine adipose cell, (**B**) the oil Red O staining of bovine adipose cells. Original magnification: ×400.

We first evaluated morphological changes in induced adipogenic bovine intramuscular adipocytes, and then stained cells with Oil Red O to visualize lipid droplet accumulation after induction. As shown in Fig. 1A, B, induced adipocytes were large, round, and filled with fat droplets, compared to immature adipocytes.

### DEG detection

**Figure 2:**
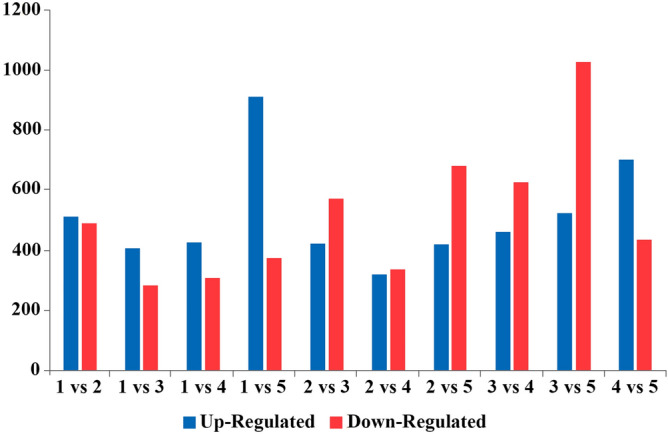
Summary of DEGs. The X axis shows the compared samples. The Y axis shows DEG numbers. The red color represents upregulated DEGs, and the blue color represents downregulated DEGs. (1) Adipogenic group (control group), (2) adipogenic + overexpression NC, (3) adipogenic + HMGR overexpression group, (4) adipogenic + interference NC, and (5) adipogenic + HMGR interference group. DEGs, differentially expressed genes.

We sequenced RNA from the five experimental groups using the Illumina HiSeq platform, and generated an average of 6.58 Gb for each sample. As showed in Fig. 2, after mapping sequenced reads to the reference genome and reconstructing the transcripts, we obtained 13,264 novel transcripts from all samples. Of these, 9444 were previously unknown splicing events for known genes, 862 were novel coding transcripts without any known features, and the remaining 2958 were long noncoding RNAs.

We then analyzed differential gene expression between samples. The summary of DEGs is shown in Fig. 2. There were a total of 10,234 DEGs, and this number was higher when the HMGR gene was overexpressed or suppressed. There were 1554 DEGs found in the HMGR overexpression group versus the HMGR interference group, where genes were mostly upregulated. We also found that there were more upregulated genes within DEGs between all experimental groups (Supplementary table S1; Supplementary Figure S1).

### Pathway analysis of DEGs

**Figure 3:**
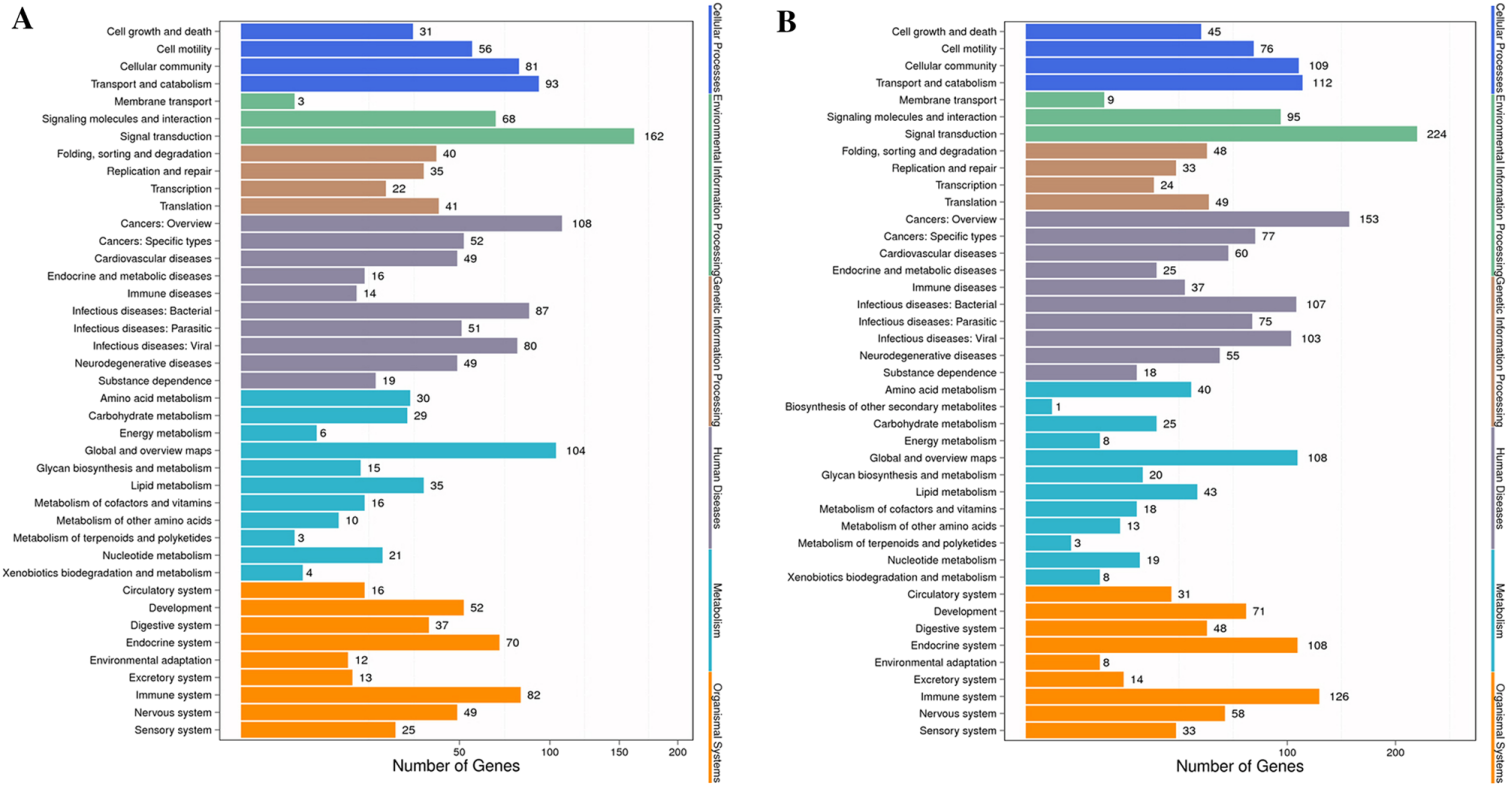
Pathway annotation of DEGs. The X axis shows the number of DEGs. The Y axis shows the pathway names. (**A)** Adipogenic group (control group) versus adipogenic + HMGR overexpression group. (**B)** Adipogenic group (control group) versus adipogenic + HMGR interference group. DEGs, differentially expressed genes.

We performed KEGG pathway classification and functional enrichment analyses for DEGs (Fig. 3). These DEGs were found to be mostly involved in signal transduction, cancers, infectious disease, global and overview maps, and the immune system. As shown in Fig. 3A, B, 35 DEGs after HMGR overexpression and 43 DEGs after HMGR inhibition, were functionally involved in lipid metabolism. Furthermore, 6 DEGs after HMGR overexpression and 8 DEGs after HMGR inhibition, were functionally involved in energy metabolism.

### AMPK signaling

**Figure 4:**
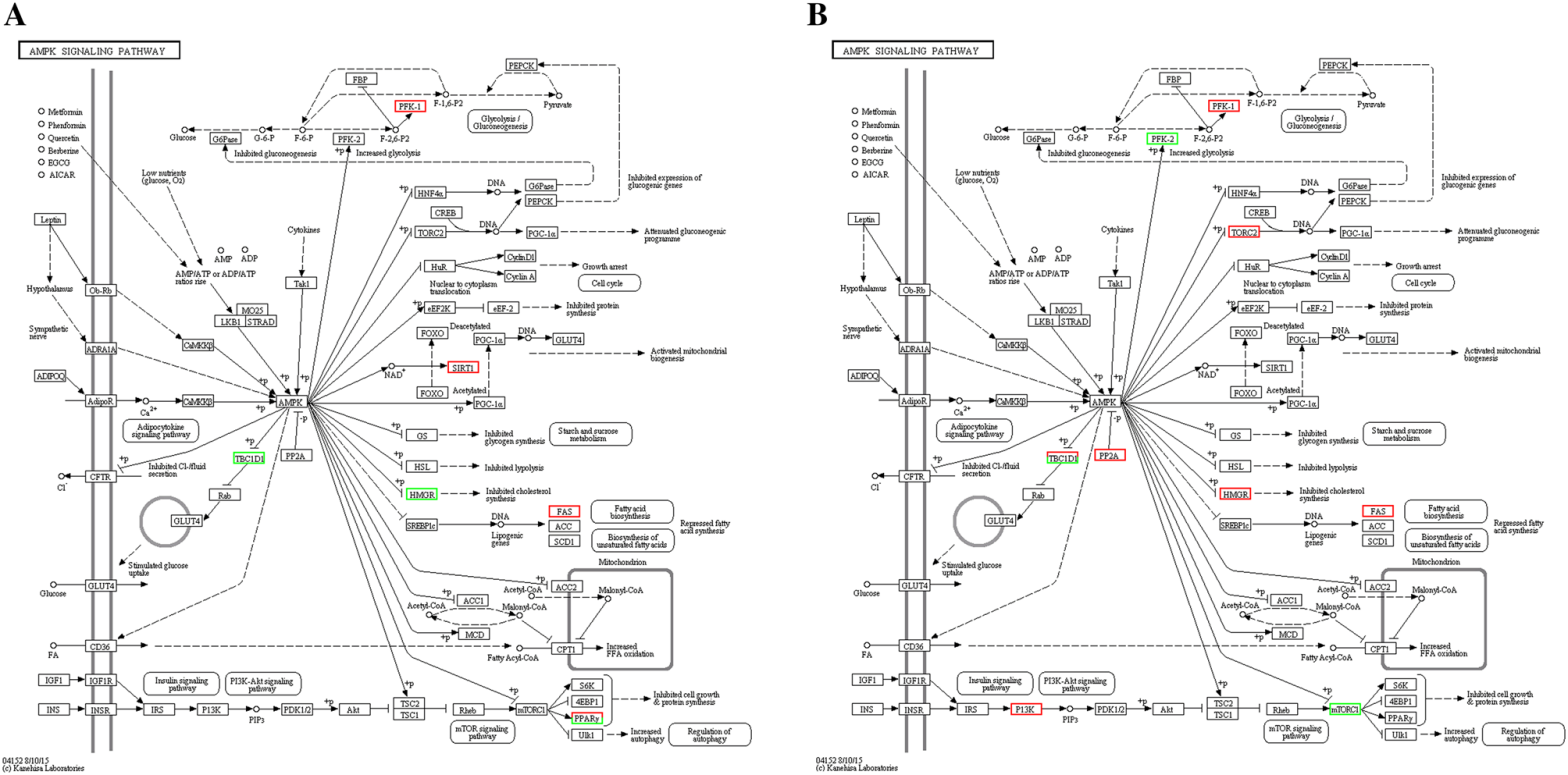
The AMPK signaling pathway. Upregulated genes are marked with red borders and downregulated genes with green borders. Non-differentially expressed genes are marked with black borders. (**A**) The AMPK signaling pathway when HMGR gene was overexpressed; (**B**) the AMPK signaling pathway when HMGR gene was interfered.

Changes in gene expression relevant to the AMPK signaling pathway following the over-expression and interference of the HMGR gene are shown in Fig. 4A, B. Interestingly, the HMGR gene was downregulated when HMGR was overexpressed, whereas the SIRT1 gene was upregulated. In contrast, the HMGR gene itself was upregulated following HMGR interference.

### TC, LDL-C, and HDL-C content in adipose cells

**Figure 5:**
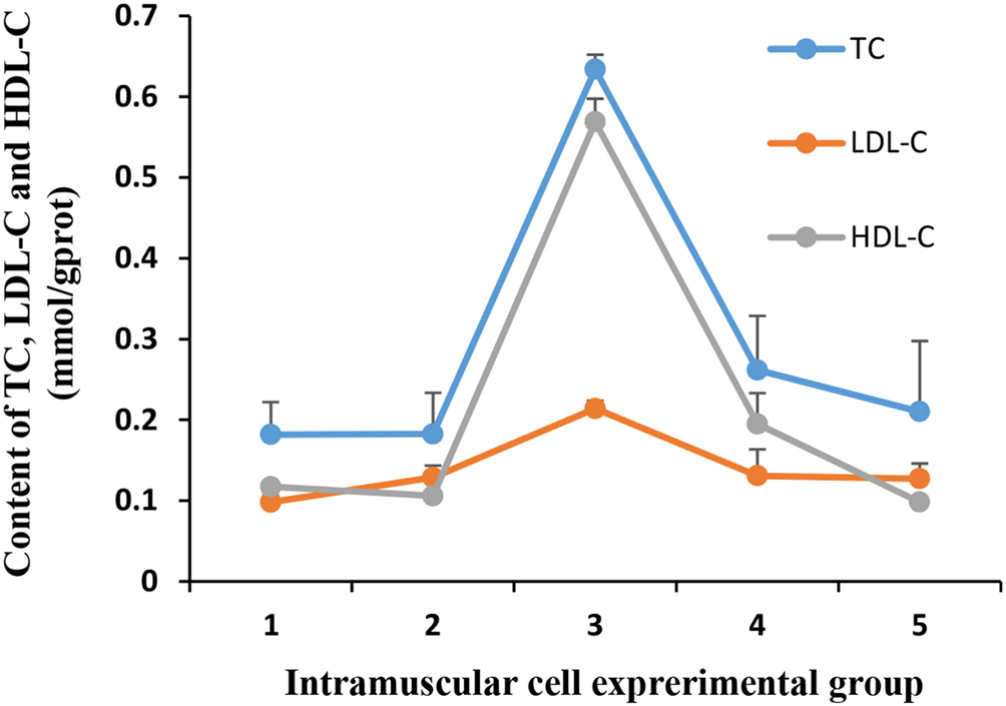
The levels of TC, LDL-C, and HDL-C in five different experimental groups. (1) Adipogenic group (control group), (2) adipogenic + overexpression NC, (3) adipogenic + HMGR overexpression group, (4) adipogenic + interference NC, and (5) adipogenic + HMGR interference group. NC, negative control. The X axis shows the groups of experiment, The Y axis shows the content of TC, LDL-C and HDL-C in cells of five different groups.

To understand the impact of HMGR overexpression on the amount of TC, LDL-C, and HDL-C, we examined the levels of these types of cholesterol in the five experimental groups. In contrast with other groups, the HMGR overexpression group had the highest amounts of TC, LDL-C, and HDL-C, particularly for TC and HDL-C (Fig. 5).

### qRT-PCR of HMGR, AMPK, SIRT1, and LPL

**Figure 6:**
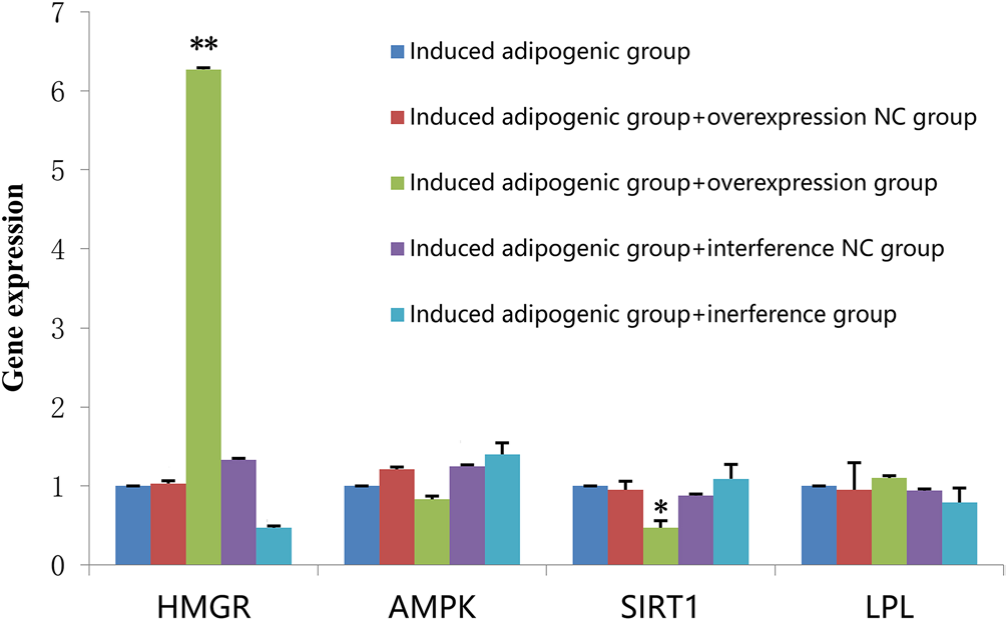
Gene expression of HMGR, AMPK, SIRT1, and LPL in intramuscular adipose cells. The X axis shows four genes, The Y axis shows the content of genes expression in cells of five different groups.

To investigate the relationship between HMGR expression and cholesterol synthesis in bovine adipose cells, the expression levels of HMGR, AMPK, SIRT1, and LPL, which are four steroidogenesis-related genes, were assessed in adipose tissue using qRT-PCR (Fig. 6).

**Figure 7:**
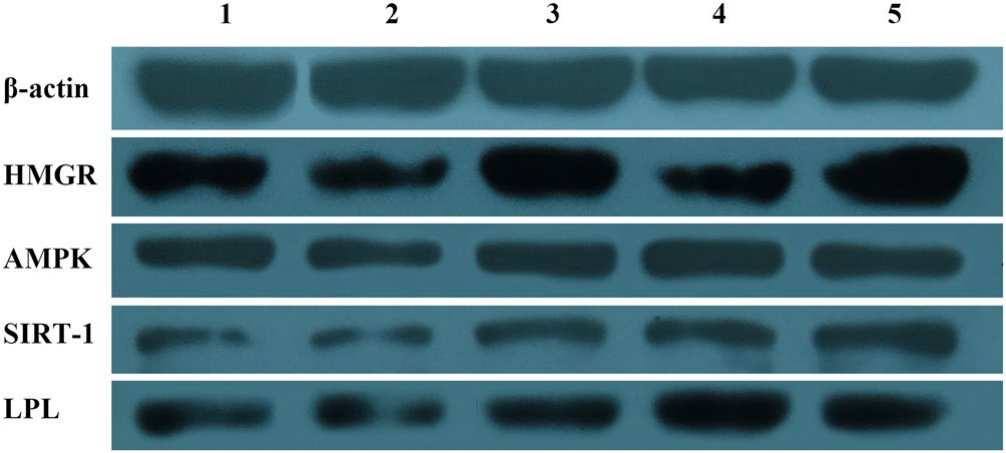
Western blotting of HMGR, AMPK, SIRT1, and LPL in the five experimental groups. Column 1: adipogenic group (control group), column 2: adipogenic group + overexpression NC, column 3: adipogenic + HMGR overexpression group, column 4: adipogenic + interference NC, and column 5: adipogenic + HMGR interference group. *NC* negative control.

For HMGR, the overexpression group showed higher mRNA expression levels than those in other groups, with the HMGR interference group showing the lowest levels out of all groups. AMPK mRNA expression was the lowest when HMGR was overexpressed; even though AMPK protein expression levels were increased (as shown in Fig. 7). SIRT1 showed similar results as AMPK, whereas LPL expression levels were higher when HMGR was overexpressed and lower when HMGR was inhibited.

### Western blotting of HMGR, AMPK, SIRT1, and LPL

To investigate changes in protein expression, we performed western blotting in the five experimental groups (Fig. 7). The results showed that HMGR protein expression in the HMGR overexpression group was higher than that in the other four groups. AMPK protein expression levels showed little differences between the groups. SIRT-1 and LPL protein expression levels were higher in the HMGR interference group and lower in the HMGR overexpression group.

## Discussion

Adipose tissue is the major depot for energy and cholesterol storage, with most of the intracellular cholesterol distributed in the form of lipid droplets^13,14^. Cholesterol homeostasis may have a role in the regulation of adipocyte size and function^15,16^. In addition, cellular cholesterol homeostasis is involved in various diseases of nongenetic origin, such as atherosclerosis, leading to lipid accumulation in target organs^15,17^.

In the present study, DEG and KEGG pathway analyses were performed to explore the regulatory network underlying bovine adipose cells following HMGR overexpression and interference. As expected, several well-known pathways^18^ related to lipid metabolism were identified, particularly the AMPK signaling pathway. In AMPK signaling, the HMGR, AMPK, and SITR1 genes play a functional role in lipid metabolism or cholesterol biosynthesis.

In terms of cholesterol levels, we found that TC, LDL-C, and HDL-C levels were the highest when the HMGR gene was overexpressed; however, their levels were not majorly decreased when HMGR was inhibited. When we investigated the protein expression of HMGR, AMPK, SIRT-1, and LPL, we found that HMGR protein levels were higher in the HMGR overexpression group compared to the control group. However, SIRT-1 protein levels were lower in the HMGR overexpression group compared to those in the HMGR interference group. Therefore, the results obtained for protein expression were consistent with those obtained for gene expression.

Given that HMGR is a rate-limiting enzyme in cholesterol biosynthesis^10^, we generated an adipocyte gene expression dataset using RNA-Seq, and examined the relationship between steroidogenic genes and cholesterol levels by overexpressing or inhibiting the HMGR gene. Transcriptional and pathway analysis results showed that the overexpression of HMGR was correlated with the downregulation of AMPK and SIRT1 gene expression.

AMPK is a crucial energy sensor that maintains energy homeostasis. It is also a major regulator of the lipid metabolism through the phosphorylation and inactivation of numerous metabolic enzymes, including HMGR^19,20^. In adipose tissue, the chronic activation of AMPK led to the downregulation of HMGR expression in a new transgenic mouse model^21^. The 5-aminoimidazole-4-carboxamide (AICA) ribonucleotide was shown to induce the activation of AMPK and directly inhibit the expression of HMGR, one of its target genes^19^. In the present study, HMGR mRNA expression was reduced when it was overexpressed in bovine adipose cells. In contrast, HMGR expression was increased when the HMGR gene was inhibited. These results are important, given the key role of the HMGR gene in the process of cholesterol synthesis.

SIRT1 is a longevity-associated deacetylase enzyme that modulates metabolic homeostasis in response to cellular energy. AMPK and SIRT1 are related proteins and share common target pathways^22^. The present study showed that the expression of SIRT1 decreased when the HMGR gene was overexpressed. However, SIRT1 was upregulated when HMGR expression was modified. These results imply that the HMGR and SIRT1 genes have contrasting roles in the cholesterol synthesis pathway.

The LPL gene encodes a rate-limiting enzyme that has a key role in the hydrolysis of triglycerides. LPL deficiency and dysfunction is associated with many disorders of lipoprotein metabolism^23,24^. The results of the present study showed that the patterns of LPL gene expression were consistent with those of HMGR expression.

## Material and methods

### Ethics statement

This study strictly followed the principles of animal use laid out by the China Laboratory Animal Science Association. The animals used in this study were reared and sacrificed in compliance with national regulations for the humane care and use of animals in research (China Administration Rule of Laboratory Animals, Operating Procedure of Cattle Slaughtering GB/T 19477-2004). All procedures using experimental animals were performed in strict accordance with the guidelines (IACC20060101, 1 January, 2006) of the Institutional Animal Care and Use Committee of Institute of Animal Science and Veterinary Medicine, Shandong Academy of Agricultural Sciences. All experimental protocols were approved by the Academic Committee of the Institute of Animal Science and Veterinary Medicine, Shandong Academy of Agricultural Sciences.

### Cell culture and induction of preadipocyte differentiation

Lilu cattle were raised in the Shandong Provincial Testing Center of Beef Cattle Performance (Shandong Province, Jinan, China). Cells from intramuscular fat tissue of the cattle were isolated according to the method described by Liu et al.^13^. The cells were cultured and maintained in DMEM/F12 (HyClone) containing 10% fetal bovine serum (Gibco) and an antibiotic–antimycotic agent (100 IU/mL penicillin and 100 μg/mL streptomycin; Gibco). Cells were seeded at 2 × 10^4^ cells/cm^2^ and incubated at 37 °C in a humidified 5% CO_2_ environment. To induce adipogenesis, two-day post-confluent preadipocytes (designated as day zero) were incubated for 72 h in differentiation-inducing medium with DMEM/F12, 4 μg/mL insulin (Sigma), 0.1 μg/mL dexamethasone (Sigma), and 0.1 mg/mL 3-isobutyl-1-methylxanthine (Sigma). The cells were then transferred into DMEM/F12 containing 1 mM octanoate (Sigma), 10 mM acetate (Sigma), 10 μg/mL transferrin (Sigma), 3 μg/mL cholesterol (Sigma), 17 mM biotin (Sigma), 100 mM calcium pantothenate (Sigma), and 0.5% bovine serum albumin (BSA). After induction, the experimental cell groups were defined as follows: (1) adipogenic group (control group), (2) adipogenic + overexpression negative control (NC) group (viral transduction control group), (3) adipogenic + HMGR overexpression group, (4) adipogenic + interference NC group (transfected with an unrelated sequence), and (5) adipogenic + HMGR interference group.

### Experimental animals

The animals were slaughtered at a slaughterhouse, Sishui Xinlv Food Co., Ltd., in Shandong Province, and permission was obtained from this slaughterhouse to use animal adipose tissues.

### Oil Red O staining

To examine lipid accumulation, cells were seeded in six-well culture plates at 5 × 10^4^/cm^2^. At eight days of incubation, the medium was removed, and the cells were washed three times with phosphate-buffered saline (Beijing Dingguo Changsheng Biotechnology Co. Ltd.) and fixed with 10% formaldehyde for 30 min at room temperature. After cells were washed with PBS, they were stained for at least 1 h with 1% filtered Oil Red O (6:4 ratio of Oil Red O stock solution to H_2_O; the Oil Red O stock solution comprised 0.5% Oil Red O in isopropyl alcohol). Morphological features of cells were examined by microscopy (Olympus Corporation).

### Construction of the HMGR expression vector

To determine the effects of HMGR on cholesterol synthesis, bovine intramuscular adipocytes were transfected with recombinant adenoviral vectors (Beijing Dingguo Changsheng Biotechnology Co. Ltd.). The recombinant vector ADV4-HMGR was then transfected into adipose cells by the method described by Hofgen and Willmitzer^25^.

### siRNA transfection

We selected four pairs of HMGR siRNAs (siRNA1, siRNA2, siRNA3, and siRNA4) for the transfection experiment, the fluorescence experiment, and the quantitative experiment to detect the transfection efficiency. The transfection efficiencies of siRNA1, siRNA2, siRNA3 and siRNA4 were found to be 46.8%, 63.8%, 46.8% and 19.1, respectively. Therefore, we selected the siRNA2 for further experiments, and the sequences of its primers as follows: sense, 5′-GUUCUAACUCACAGGAUGATT-3′; and antisense, 5′-UCAUCCUGUGAGUUAGAACTT-3′. The siRNA2 oligonucleotides were designed by Beijing Dingguo Changsheng Biotechnology Co. Ltd.. The siRNA2 inhibition group and siRNA2 negative control group were transfected for 48 h using the lipofectamine transfection reagent (Invitrogen), according to the manufacturer's protocol.

### Detection of DEGs

The generation and processing of raw RNA-seq data were conducted by the Beijing Genomics Institute using the Illumina HiSeq platform. We detected DEGs using the PossionDis algorithm, which is based on the Poisson distribution, as described by Audic et al.^26^. The parameters to determine significant changes in gene expression were as follows: fold change ≥ 2 and false discovery rate (FDR) ≤ 0.001.

### Pathway analysis of DEGs

DEGs were annotated according to the Kyoto Encyclopedia of Genes and Genomes (KEGG) database. We performed pathway enrichment analysis using phyper, a function in R based on hypergeometric distribution^27,28^. We then calculated the FDR for each p-value, and considered an FDR < 0.001 to represent significant enrichment.

### Quantification of total cholesterol in adipose cells

Total cholesterol (TC), high-density lipoprotein cholesterol (HDL-C), and low-density lipoprotein cholesterol (LDL-C) levels were measured in adipose cells after seeding in a six-well culture plate using detection kits specific to each cholesterol type (T-CHO kit, HDL-C kit, and LDL-C kit; Nanjing Jiancheng Biology Institute).

### Total RNA isolation and quantitative real-time PCR

Total RNA was isolated from adipose cells using the TRIzol reagent (Invitrogen), and then converted into cDNA using the PrimeScript RT Reagent Kit (TaKaRa). Expression of selected genes was then analyzed using the Fast SYBR Green Master Mix Bulk Pack (Invitrogen). The following primers were used for quantitative real-time PCR (qRT-PCR): HMGR sense, 5′-TGCTGGTGCTGAGTATGTGG-3′ and antisense, 5′-CATTCTACAAGAGCATCCAGG-3′; LPL sense, 5′-GACACTTGCCACCTCATTC-3′ and antisense, 5′-CATCCGCCATCCAGTTCATA-3′; AMPK sense, 5′-CCGTATTATTTGCGTGTTCG-3′ and antisense, 5′-TGTGGCGTAGCAGTCCCT-3′; SIRT1 sense, 5′-TGCAATAGACTTTCCAGACC-3′ and antisense, 5′-GTGTATCTATGTTCTGAGT-3′; and GAPDH sense, 5′-TGCTGGTGCTGAGTATGTGG-3′ and antisense, 5′-GATGATGACCCTCTTGGCG-3′ (Beijing Dingguo Changsheng Biotechnology Co. Ltd.). Three replicates were used for each experiment. Relative mRNA expression levels were measured using the comparative Ct method (∆∆Ct), with GAPDH as the internal control.

### Western blotting

Cell extracts were isolated in RIPA buffer (1 mL per 10^7^ cells/100 mm dish/150 cm^2^ flask; 0.5 mL per 5 × 10^6^ cells/60 mm dish/75 cm^2^ flask; WB-0071; Beijing Dingguo Changsheng Biotechnology Co. Ltd.). The Pierce BCA Assay (Beijing Dingguo Changsheng Biotechnology Co. Ltd.) was used to determine protein concentration. Samples (50–100 μg) were separated using 10% sodium dodecyl sulfate polyacrylamide gel electrophoresis, and then transferred to polyvinylidene fluoride membranes (Pall). The membranes were incubated with anti-β-actin (Santa Cruz Biotechnology, Inc.), anti-HMGR (Abcam), anti-AMPK (Abcam), anti-LPL (Abcam), and anti-SIRT1 (Bioss Biotechnology, Inc.) antibodies in blocking buffer (5 g non-fat milk power, 100 mL TBST buffer (0.05% Tween 20, 1.65 mL + TBS, 700 mL, pH7.5)) for 2 h at room temperature. After the membranes were incubated with HRP-labeled goat Anti-rabbit IgG and HRP-labeled goat anti-Mouse IgG (Beijing Dingguo Changsheng Biotechnology Co. Ltd.), the enhanced ECL chemiluminescence detection kit (Pierce) was used to detect immunoreactive bands.

### Statistical analysis

Differences in gene expression between the experimental groups were calculated by analysis of variance (ANOVA), followed by the Bonferroni test. Results were considered statistically significant at *P* < 0.05.

## Supplementary information

Supplementary information.

## Data Availability

The datasets generated and/or analyzed during the current study are available from the corresponding author on reasonable request.
