# Supplementary material for: HMGR overexpression and interference affect the expression of steroidogenic genes and cholesterol content in bovine intramuscular adipocytes
Source: Sci Rep. 2020 Oct 6;10:16606. doi: 10.1038/s41598-020-73626-8 (PMC7538946; doi:10.1038/s41598-020-73626-8)
Supplement: Supplementary file 1 — Supplementary information. [file 41598_2020_73626_MOESM1_ESM.docx]

**Title:**

**HMGR overexpression and interference affect the expression of steroidogenic genes and cholesterol content in bovine intramuscular adipocytes**

**Authors:**

Haichao Lin^1,2,3,+^, Chen Wei^1,2,3, +^, Xianglun Zhang^1,2,3^, Wei You^1,2,3^, Qing Jin^1,2,3^, Xiuwen Tan^1,2,3^, Hongbo Zhao^1,2,3^, Chen Zhang^4^, Xiaomu Liu^1,2,3^ & Guifen Liu^1,2,3,+*^

**Affiliations:**

1 Institute of Animal Science and Veterinary Medicine, Shandong Academy of Agricultural Sciences, Jinan, 250100, China

2 Shandong Key Lab of Animal Disease Control and Breeding, Jinan, 250100, China

3 Shandong Provincial Testing Center of Beef Cattle Performance, Jinan, 250100, China

4 College of Life Sciences, Shandong Normal University, Jinan, 250100, China

* Correspondence: Guifen Liu, [liuguifen126@126.com](#mailto:wanfc@sina.com)

Postal address: lichen region, jinan, 250100, China

Tel: +86053188622249

^+^ These authors contributed equally to this work.

1 2 3 4 5


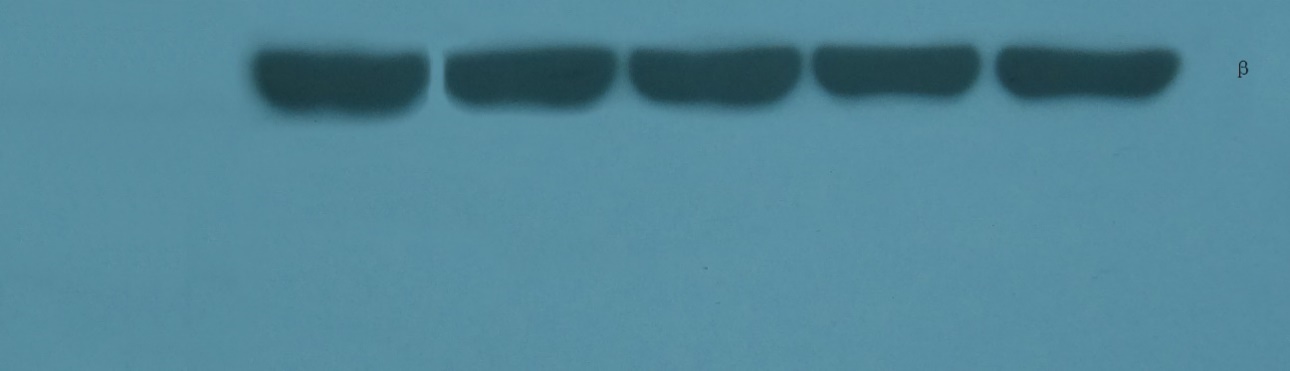


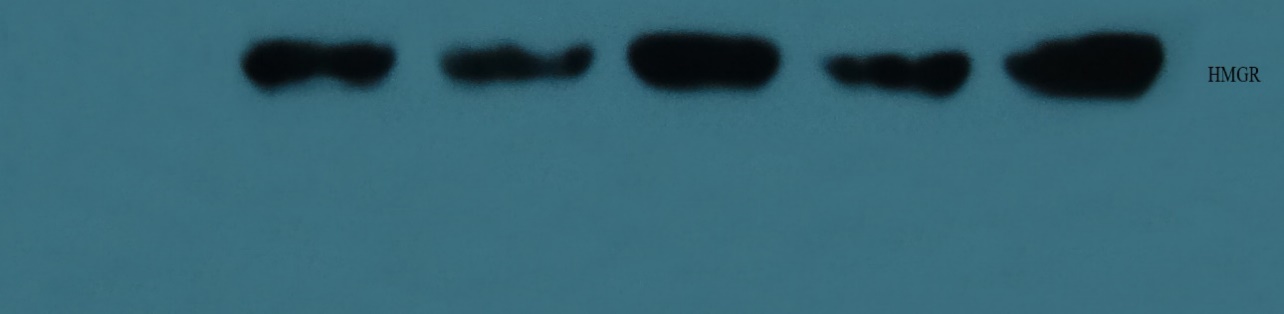


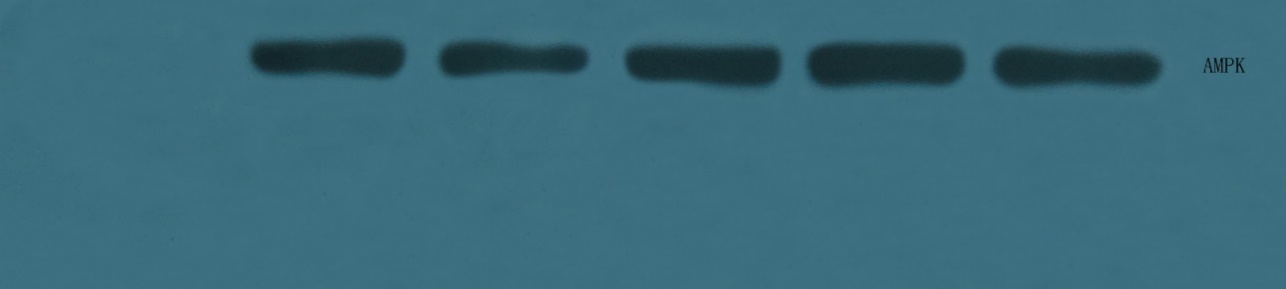


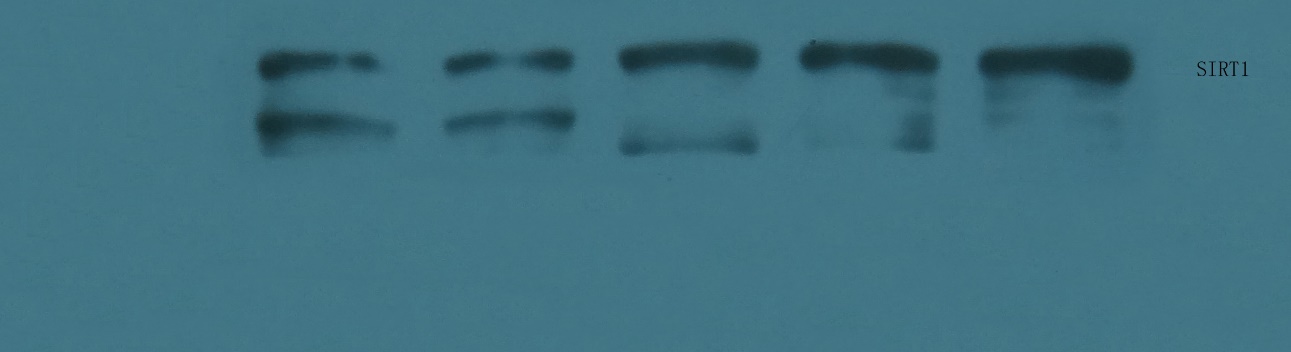


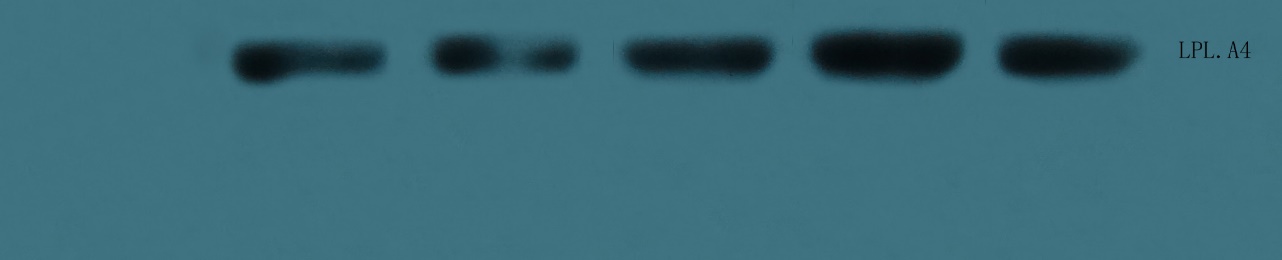


**Supplementary Figure S1.** Western blotting of HMGR, AMPK, SIRT1, and LPL in the five experimental groups. Column 1: adipogenic group (control group), column 2: adipogenic group + overexpression NC, column 3: adipogenic + HMGR overexpression group, column 4: adipogenic + interference NC, and column 5: adipogenic + HMGR interference group. NC, negative control.

**Supplementary table S1.** Analysis of the optical density of the Western blot bands with Quantity One.

| Sample  optical  density | Column 1 | Column 2 | Column 3 | Column 4 | Column 5 |
| --- | --- | --- | --- | --- | --- |
| optical density of β-actin | 13862.58 | 11925.61 | 12001.59 | 10605.91 | 11085.92 |
| optical density of AMPK | 5010.484 | 3700.835 | 5204.016 | 5941.268 | 5008.935 |
| AMPK /β-actin | 0.36144 | 0.310327 | 0.433611 | 0.560184 | 0.451829 |
| optical density of HMGR | 7174.998 | 5065.188 | 9006.536 | 5789.226 | 9947.753 |
| HMGR /β-actin | 0.51758 | 0.424732 | 0.750445 | 0.545849 | 0.897332 |
| optical density of LPL | 4351.788 | 3746.186 | 4984.198 | 7174.108 | 5234.567 |
| LPL /β-actin | 0.313923 | 0.314129 | 0.415295 | 0.676425 | 0.472182 |
| optical density of SIRT1 | 6129.865 | 3948.54 | 4983.935 | 5069.438 | 7174.556 |
| SIRT1/β-actin | 0.442188 | 0.331097 | 0.415273 | 0.477982 | 0.647177 |
